# Ethnic background and children’s television viewing trajectories: The Generation R Study

**DOI:** 10.1371/journal.pone.0209375

**Published:** 2018-12-14

**Authors:** Junwen Yang-Huang, Amy van Grieken, Lu Wang, Vincent W. V. Jaddoe, Wilma Jansen, Hein Raat

**Affiliations:** 1 The Generation R Study Group, Erasmus Medical Center, Rotterdam, The Netherlands; 2 Department of Public Health, Erasmus Medical Center, Rotterdam, The Netherlands; 3 Department of Pediatrics, Erasmus Medical Center, Rotterdam, The Netherlands; 4 Department of Epidemiology, Erasmus Medical Center, Rotterdam, The Netherlands; McMaster University, CANADA

## Abstract

This study, conducted in the Netherlands, evaluated the association between ethnic background and children’s TV viewing time at multiple time points and its trajectory. We analyzed 4,833 children with a Dutch, Moroccan, Turkish, or Surinamese ethnic background from the Generation R Study, a population-based study in the Netherlands. Parent-reported television viewing time for children at ages 2, 3, 4, 6, and 9 years was collected by questionnaires sent from April 2004 until January 2015. Odds ratios of watching television ≥1 hour/day at each age were calculated for children from the various ethnic backgrounds. Generalized logistic mixed models (GLMMs) were used to assess the association between ethnic background and television viewing time trajectory. The effect modification by family socioeconomic status was examined in cross-sectional and longitudinal analyses. The percentage of children viewing television ≥1 hour/day increased from age 2 to 9 years for children from all ethnic backgrounds. After adjusting for maternal educational level and net household income, children from all ethnic subgroups had greater odds of watching television ≥1 hour/day at some time points compared with children with a Dutch background (Surinamese: all ages; Moroccan: at ages 4 and 6 years; Turkish: at ages 4 and 9 years). The GLMMs indicated that television viewing trajectories differed between ethnic subgroups. The associations between ethnic background and children’s television viewing time were moderated by maternal educational level for child ages 4 and 6 years (p < 0.05). In longitudinal analyses, the ethnic differences in probability of watching television ≥1 hour/day were larger in children from high-educated mothers than in children from low-educated mothers. In conclusion, ethnic differences in television viewing time were present at all measuring time points. The discrepancy between children with a Dutch background and children with another background was larger in high maternal educational subgroups.

## Introduction

Multiple studies have shown that excessive screen time contributes to the risk of childhood overweight and obesity [1]. Currently, health authorities in Australia and Canada recommend children aged 5–12 years spend no more than 2 hours a day on electronic media for entertainment [2, 3]. For children 2 to 5 years of age, the American Academy of Pediatrics advises limiting screen use to 1 hour per day [4]. Nonetheless, there remains room for improving the screen use behavior in most young children [5, 6]. Despite the development of new media technologies over the past 10 years, television (TV) viewing is still the predominant screen time behavior for young children [7]. Such early life behavior has been shown to persist across age, indicating that children spending more time viewing TV when they are young are more likely to spend more time viewing TV later in adolescence [8, 9]. Children who spend much time on TV viewing also spend more time using other media such as video games, computers, and smartphones [10]. A study investigating different sedentary behaviors found that they influenced child health differently and that TV viewing was most strongly related to the overweight development [11]. The increasing amount of time children spent viewing TV and the potential implications of TV viewing on health outcomes highlight the importance of preventing excessive TV viewing behavior in early life.

Ethnic differences in childhood overweight have been reported in several country settings [12, 13]. In the Netherlands, the prevalence of overweight and obesity is much higher among children with a Turkish and Moroccan background than among native Dutch children [14]. Furthermore, studies in other countries have revealed that ethnic minority groups spend more time watching TV than their native counterparts [15, 16]. However, little is known about the differences in TV viewing time between children from different ethnic backgrounds in the Netherlands [17]. Moreover, longitudinal studies on the development of TV viewing time among children with different ethnic backgrounds may provide important information to policy makers and researchers regarding the optimal age as well as which groups to target with preventive interventions aimed at reducing TV viewing time.

Our study had three aims. The first was to evaluate the associations between ethnic background and children’s TV viewing time at multiple time points: ages 2, 3, 4, 6, and 9 years. We hypothesized that children with a non-Dutch ethnic background would have higher TV viewing time than children with a Dutch background. The second aim was to assess the association between ethnic background and children’s TV viewing trajectory, i.e., TV viewing time over multiple time points. We hypothesized that TV viewing trajectories would differ between subgroups with a different ethnic background. The third aim was to examine to what extent the associations between child ethnic background and TV viewing time were modified by socioeconomic differences. On the basis of our previous findings [17], we hypothesized that there would be significant effect modification by family socioeconomic status (SES) (i.e., maternal educational level).

## Methods

### Study design

This study was embedded in the Generation R Study, a population-based prospective cohort study from fetal life until young adulthood in the Netherlands. Pregnant women with an expected delivery date between April 2002 and January 2006 living in Rotterdam were eligible for participation in the study [18]. Extensive assessments are performed in mothers, fathers, and their children. The study was conducted in accordance with the guidelines proposed in the World Medical Association’s Declaration of Helsinki and was approved by the Medical Ethical Committee at Erasmus Medical Center, University Medical Center Rotterdam. Written informed consent was obtained from all participants.

### Study population

In total, 9,778 mothers were enrolled in the study and gave birth to 9,745 known live born children. Consent for postnatal follow-up during the preschool period (0–4 years) and/or the school period (6–9 years) was available for 9,162 children. For the purpose of this study, we selected children with Dutch, Moroccan, Turkish, or Surinamese ethnic backgrounds (n = 6,497). These subgroups were chosen because they represent the largest ethnic background in the Generation R Study as well as in the city of Rotterdam [19]. We excluded participants with missing information on TV viewing at all measuring time points (n = 1,209). To avoid clustering of data, we further excluded second (n = 446) and third children (n = 9) of the same mother, leaving a study population of 4,833 participants. The final population for analysis consisted of 3,561 children with a Dutch background, 317 children with a Moroccan background, 498 children with a Turkish background and 457 children with a Surinamese background (see S1 Appendix).

### Ethnic background

The ethnic backgrounds of the mothers and children were categorized with the standard methods used in the Netherlands [20]. The children were assigned to the subgroups as their mothers’ ethnic background with the cultural background of the mothers is taken into account. Maternal ethnic background was based on the country of birth of the mother and of her parents; this information was obtained by the questionnaire completed at enrollment. In accordance with Statistics Netherlands, if the mother was born outside the Netherlands, this country of birth determined the ethnic background. If she was born in the Netherlands, but one of her parents was born outside the Netherlands, this country of birth determined the ethnic background. If both her parents were born outside the Netherlands, her mother’s country of birth determined her ethnic background.

### TV viewing time

Children’s TV viewing time was assessed at five time points (child ages 2, 3, 4, 6, and 9 years) by parent-reported questionnaires. Parents were asked about the average number of days per week or/and weekend their child spent viewing TV. Subsequently, they were asked to indicate the average time spent per day viewing TV. We took the duration of TV viewing per session to be the median number of hours (e.g., 1.5 hours in the case of “1–2 hours”). The average TV viewing time per day was derived by multiplying the duration per day by the number of days of TV viewing, which was then divided by seven. Week and weekend days were combined. TV viewing time for children aged 2 or 3 was obtained differently from that of children of other ages, because the number of days of TV viewing was not available for these very young children. To harmonize the measurements, we estimated average TV time for children aged 2 and 3 years from the number of days of TV viewing at age 4 years. 54% of parents indicated that at that age their children watched TV seven days per week. More information on the TV viewing time is available in S1 Table and in an earlier publication [21]. Based on the latest recommendation from the American Academy of Pediatrics [4], TV viewing time was dichotomized at more than or equal to 1 hour per day. Sensitivity analyses using a secondary outcome variable dichotomized at 2 hours per day were also performed [2, 3]. The results are available in S2 Table.

### Potential confounders

Child gender, age, marital status, and family SES were considered potential confounders in the associations between ethnic background and children’s TV viewing time. Child age was obtained by questionnaires at each measuring time point. Mothers’ marital status was assessed by questionnaires at enrollment and dichotomized as married/cohabiting or no partner. Family SES was captured by two following indicators: maternal educational level and net household income. The highest educational level attained by the mother was established using questionnaires at enrollment. The Dutch Standard Classification of Education was used to categorize three levels of education: low (no education, primary school, lower vocational training, intermediate general school, or 3 years or less general secondary school); middle (>3 years general secondary school, intermediate vocational training); and high (higher vocational training, university or PhD degree) [22]. Net household income was assessed by questionnaire at enrollment and classified into two categories: high (>€2,200 per month) and low (less than €2,200 per month) [23]. Sensitivity analyses were performed using paternal educational level as measurement of family SES. The results are available in S3 Table.

### Statistical analyses

First, frequency tables and cross tabulations were used to explore characteristics of the study population. The association between child ethnic background and TV viewing time at each measuring time point was assessed cross-sectionally using logistic regression models. Child age, maternal educational level, and net household income led to a substantial change in effect estimates (i.e., ≥10% change) and were included in the models as confounders [24]. Odds ratios (ORs) and 95% confidence intervals (CI) are reported for each subgroup compared to the reference group of children with a Dutch background. A significance level of p<0.05 was taken to indicate a significant association between ethnic background and children’s TV viewing time. A multiple imputation procedure was used to impute missing values in the covariates (ranging from 0% to 28.2%, see Table 1). Five imputed datasets were generated using a fully conditional specified model, based on the relationships between all the variables included in this study [25]. Pooled estimates from these five imputed datasets were used to report ORs and their 95% CI. Cross-sectional analyses were performed on both the non-imputed and imputed datasets and the results were found to be comparable.

**Table 1:** Characteristics of the total study population and per ethnic subgroup (n = 4,833)^a^.

		Ethnic Background				
	Total	Dutch	Turkish	Moroccan	Surinamese	P-value^b^
	N = 4,833	n = 3,561	n = 498	n = 317	n = 457	
		(73.7%)	(10.3%)	(6.6%)	(9.5%)	
**Family Characteristics**^**c**^						
Maternal educational level (%)						
Low	20.3	12.8	52.5	47.1	30.4	<0.001
Middle	29.8	26.4	34.4	36.6	48.6	
High	49.9	60.8	13.1	16.3	21.0	
Maternal age years (SD)	31.0 (4.9)	31.9 (4.4)	27.8 (5.1)	29.0 (5.2)	28.9 (5.5)	<0.001
Marital status (%)						
Married/cohabiting	91.8	94.2	93.9	96.7	67.4	<0.001
No partner	8.2	5.8	6.1	3.3	32.6	
Net Household Income (%)						
Less than €2200/month	37.7	25.0	86.9	89.0	64.2	<0.001
> €2200/month	62.3	75.0	13.1	11.0	35.8	
**Child Characteristics**^**c**^						
Gender (%)						
Boy	51.1	50.4	52.6	50.2	55.6	0.18
Child’s exact age Mean (SD)						
Age 2 years	24.4 (1.1)	24.4 (1.1)	24.6 (1.3)	24.8 (1.3)	24.6 (1.3)	<0.001
Age 3 years	36.6 (1.3)	36.5 (1.1)	37.0 (1.7)	37.0 (1.8)	36.9 (1.7)	<0.001
Age 4 years	48.6 (1.1)	48.5 (1.0)	49.1 (1.6)	49.0 (1.5)	48.9 (1.2)	<0.001
Age 6 years	72.4 (5.5)	71.8 (4.8)	74.3 (6.6)	75.1 (7.8)	73.7 (6.5)	<0.001
Age 9 years	116.5 (3.7)	116.2 (3.4)	117.7 (4.2)	118.5 (5.6)	117.0 (4.0)	<0.001
TV viewing time ≥ 1 hour/day (%)						
Age 2 years	12.3	10.0	17.4	23.9	30.2	<0.001
Age 3 years	30.9	27.5	47.1	50.0	46.7	<0.001
Age 4 years	36.8	32.4	54.5	56.5	54.3	<0.001
Age 6 years	57.6	52.9	70.8	75.6	73.4	<0.001
Age 9 years	72.8	69.8	84.8	80.1	89.0	<0.001
TV viewing time ≥ 2 hour/day (%)						
Age 2 years	0	0	0	0	0	-
Age 3 years	6.0	3.9	18.6	14.9	14.3	<0.001
Age 4 years	8.6	5.5	22.0	18.2	21.8	<0.001
Age 6 years	18.2	11.6	39.1	40.3	39.7	<0.001
Age 9 years	29.5	24.2	50.5	46.8	56.1	<0.001

Table is based on non-imputed dataset.

^a^ Values are percentages or means (SD) for the total population and per ethnic subgroup.

^b^ P-values are calculated by Chi-square test for categorical variables and ANOVA for continuous variables.

^c^ Data were missing for maternal educational level (5.5%), household income (21.1%), child’s exact age at 2 (26.2%), 3 (29.6%), 4 (26.9%), 6 (9.4%) and 9 (23.7%) years and child TV viewing time at 2 (33.8%), 3 (36.4%), 4 (28.0%), 6 (15.1%) and 9 (31.9%) years.

Generalized logistic mixed models (GLMM) were used to assess the association between ethnic background and children’s TV viewing time trajectory. These longitudinal models take the correlation between repeated-measured TV viewing time into account and allow for incomplete outcome variables. The p-value (p<0.05) of the interaction effect between ethnic background and child age indicated whether socioeconomic differences changed with the age of the child.

To assess effect modification by maternal educational level and net household income, an interaction term between ethnic background and each indicator of family SES was added to the cross-sectional models. For the longitudinal models, the interaction term between ethnic background and each indicator of SES was tested in the GLMM models. Only the significant interaction terms (p<0.05) were included in the final models to show TV viewing trajectories in the various SES subgroups.

Descriptive analyses and cross-sectional analyses were performed using the Statistical Package for Social Sciences (SPSS) version 21.0 for Windows (SPSS Inc, Chicago, IL, USA) and longitudinal models were fitted using the lme4 package in R version 3.3.2 for Windows (R Foundation for Statistical Computing).

## Results

### Participant characteristics

Table 1 shows the characteristics of the study population. Over 25% of the mothers had a non-Dutch ethnic background. Compared to Dutch mothers, non-Dutch mothers were more frequently lower educated and more often had a low household income (less than €2,200/month) (all p<0.001). Compared to non-Dutch mothers, Dutch mothers more often were married or cohabiting (94.2% for Dutch mothers versus 85.0% for non-Dutch mothers, p<0.001), except for mothers with a Moroccan background (96.7%). For all ethnic subgroups of children, the percentage viewing TV ≥1 hour/day increased from ages 2 to 9 years (from 10.0% to 69.8% for children with a Dutch background; from 17.4% to 84.8% for children with a Turkish background; from 23.9% to 80.1% for children with a Moroccan background; from 30.2% to 89.0% for children with a Surinamese background). Children from all subgroups had greater odds of exceeding the recommendations on screen use of 1 hour per day compared to children with a Dutch background (all p < 0.001).

### Associations between ethnic background and children’s TV viewing time at ages 2, 3, 4, 6, and 9 years

In the total study population, ethnic background was significantly associated with TV viewing time at all ages (all p < 0.001, Table 2). After adjusting for maternal educational level and net household income, children with a Surinamese background had greater odds of watching TV ≥1 hour/day compared with children with a Dutch background at all ages, with the highest risk in the group at age 9 years (OR: 2.65, 95%CI: 1.76, 3.98). Compared with children with a Dutch background, children with a Moroccan background had greater odds of watching TV ≥1 hour/day at age 4 years (OR: 1.63, 95%CI: 1.14, 2.33) and age 6 years (OR: 1.83, 95%CI: 1.30, 2.56), children with a Turkish background had greater odds of watching TV ≥1 hour/day at age 4 years (OR: 1.46, 95%CI: 1.10, 1.95), age 6 years (OR: 1.38, 95%CI: 1.06, 1.78)and age 9 years (OR: 1.61, 95%CI: 1.06, 2.44).

**Table 2:** Associations between ethnic background and TV viewing time at each age (n = 4,833).

Ethnicity	Measuring time point				
	Age 2 years	Age 3 years	Age 4 years	Age 6 years	Age 9 years
	OR (95%CI)	OR (95%CI)	OR (95%CI)	OR (95%CI)	OR (95%CI)
Dutch	1.00	1.00	1.00	1.00	1.00
Turkish	0.93 (0.62, 1.39)	1.23 (0.89, 1.69)	**1.46 (1.10, 1.95)**	**1.38 (1.06, 1.78)**	**1.61 (1.06, 2.44)**
Moroccan	1.42 (0.88, 2.30)	1.46 (0.97, 2.20)	**1.63 (1.14, 2.33)**	**1.83 (1.30, 2.56)**	1.20 (0.78, 1.85)
Surinamese	**2.38 (1.67, 3.38)**	**1.54 (1.12, 2.13)**	**1.72 (1.30, 2.28)**	**1.83 (1.42, 2.37)**	**2.65 (1.76, 3.98)**

Table is based on imputed dataset. Bold print indicates statistical significance. Values represent odds ratios and 95% confidence intervals derived from multiple logistic regression analyses.

Models were adjusted for child’s exact age, maternal educational level, and net household income.

### Effect modifications by family SES

The associations between ethnic background and children’s TV viewing time were moderated by maternal educational level at child ages 4 and 6 years (p < 0.05). No moderation was observed for net household income. Among children from low-educated mothers, children with a Moroccan background had greater odds of watching TV ≥1 hour/day compared to children with a Dutch background. (OR: 2.14, 95%CI:1.10, 4.19) (Table 3).

**Table 3:** Associations of ethnic background with TV viewing time according to maternal educational level at each age (n = 4,833).

Maternal educational level	Ethnic background	Measuring time point				
		Age 2 years	Age 3 years	Age 4 years	Age 6 years	Age 9 years
		OR (95%CI)	OR (95%CI)	OR (95%CI)	OR (95%CI)	OR (95%CI)
High	Dutch	1.00	1.00	1.00	1.00	1.00
	Turkish	1.31 (0.39, 4.45)	2.17 (1.00, 4.71)	**3.10 (1.53, 6.24)**	1.85 (0.87, 3.91)	**2.49 (1.02, 6.07)**
	Moroccan	2.19 (0.58, 8.27)	2.28 (0.90, 5.74)	**5.21 (1.87, 14.54)**	**2.21 (1.03, 4.73)**	2.08 (0.86, 5.01)
	Surinamese	**3.77 (1.91, 7.47)**	**2.65 (1.46, 4.81)**	**3.09 (1.80, 5.32)**	**2.45 (1.47, 4.11)**	**6.74 (2.46, 18.50)**
Middle	Dutch	1.00	1.00	1.00	1.00	1.00
	Turkish	1.67 (0.96, 2.90)	1.40 (0.83, 2.35)	**1.77 (1.13, 2.79)**	**1.98 (1.27, 3.08)**	1.63 (0.85, 3.12)
	Moroccan	1.93 (0.92, 4.06)	1.49 (0.76, 2.93)	1.61 (0.89, 2.92)	1.37 (0.83, 2.26)	1.08 (0.54, 2.14)
	Surinamese	**2.73 (1.66, 4.48)**	1.40 (0.88, 2.22)	**1.62 (1.08, 2.41)**	**1.58 (1.09, 2.29)**	**1.89 (1.09, 3.28)**
Low	Dutch	1.00	1.00	1.00	1.00	1.00
	Turkish	**0.41 (0.22, 0.78)**	0.81 (0.51, 1.28)	0.78 (0.52, 1.18)	0.89 (0.60, 1.32)	1.00 (0.85, 3.12)
	Moroccan	0.82 (0.39, 1.70)	1.01 (0.53, 1.90)	0.82 (0.46, 1.46)	**2.14 (1.10, 4.19)**	0.74 (0.36, 1.53)
	Surinamese	1.23 (0.63, 2.39)	0.95 (0.51, 1.79)	0.87 (0.49, 1.53)	1.73 (0.99, 3.01)	1.64 (0.58, 4.61)

Table is based on imputed dataset. Bold print indicates statistical significance. Values represent odds ratios and 95% confidence intervals derived from multiple logistic regression analyses.

Models were adjusted for child’s exact age and net household income.

Among children from middle-educated mothers and compared to children with a Dutch background, children with a Surinamese background had greater odds of watching TV ≥1 hour/day at all ages except age 3 years; the highest risk was in the group at age 2 years (OR: 2.73, 95%CI: 1.66, 4.48). Children with a Turkish background had greater odds of watching TV ≥1 hour/day at ages 4 years (OR: 1.77, 95%CI: 1.13, 2.79) and 6 years (OR: 1.98, 95%CI: 1.27, 3.08).

Among children from high-educated mothers, children with a Surinamese background had greater odds of watching TV ≥1 hour/day compared to children with a Dutch background at all ages, with the highest risk in the group at age 9 years (OR: 6.74, 95%CI: 2.46, 18.50). The highest risk compared to children with a Dutch background was found in children with a Moroccan or Turkish background at age 4 years (for the Moroccan subgroup, OR: 5.21, 95%CI: 1.87, 14.54; for the Turkish subgroup, OR: 3.10, 95%CI: 1.53, 6.24). Sensitivity analyses using paternal educational level showed that the associations between ethnic background and children’s TV viewing time were moderated by paternal educational level at child ages 2, 3, and 6 years (p < 0.05). The results were comparable to the analyses using maternal educational level. Among children from low-educated fathers, no significant differences in children's TV viewing time were found between ethnic subgroups (S3 Table).

### Associations between ethnic background and children’s TV viewing time trajectories

**Fig 1:**
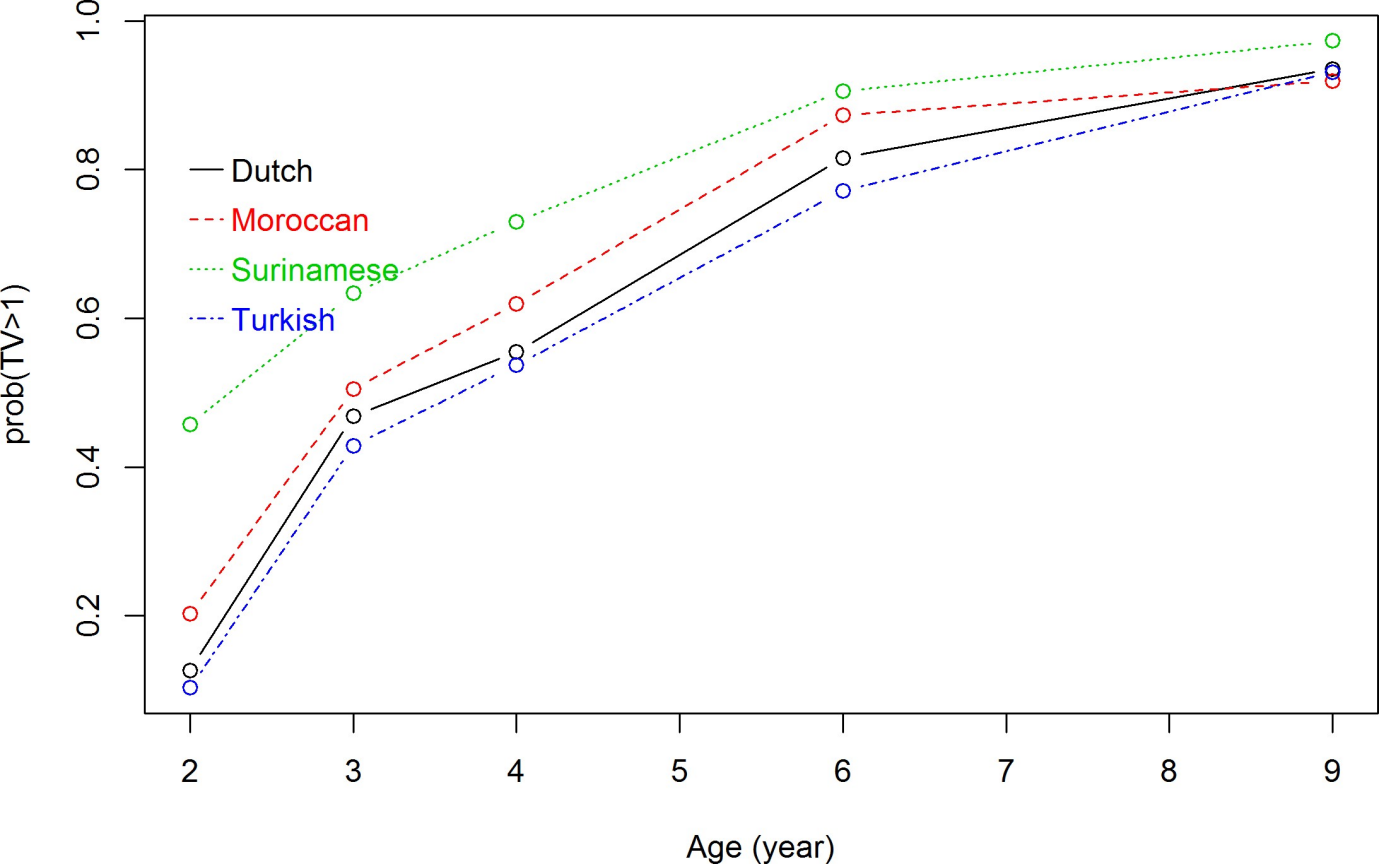
Association between ethnic background and TV viewing time trajectories in children of low-educated mothers. Results are based on a generalized logistic mixed model and reflect the probability of TV viewing time of >1 hour/day (based on 2,579 measurements) in the first 9 years for children of mothers with low education level.

**Fig 2:**
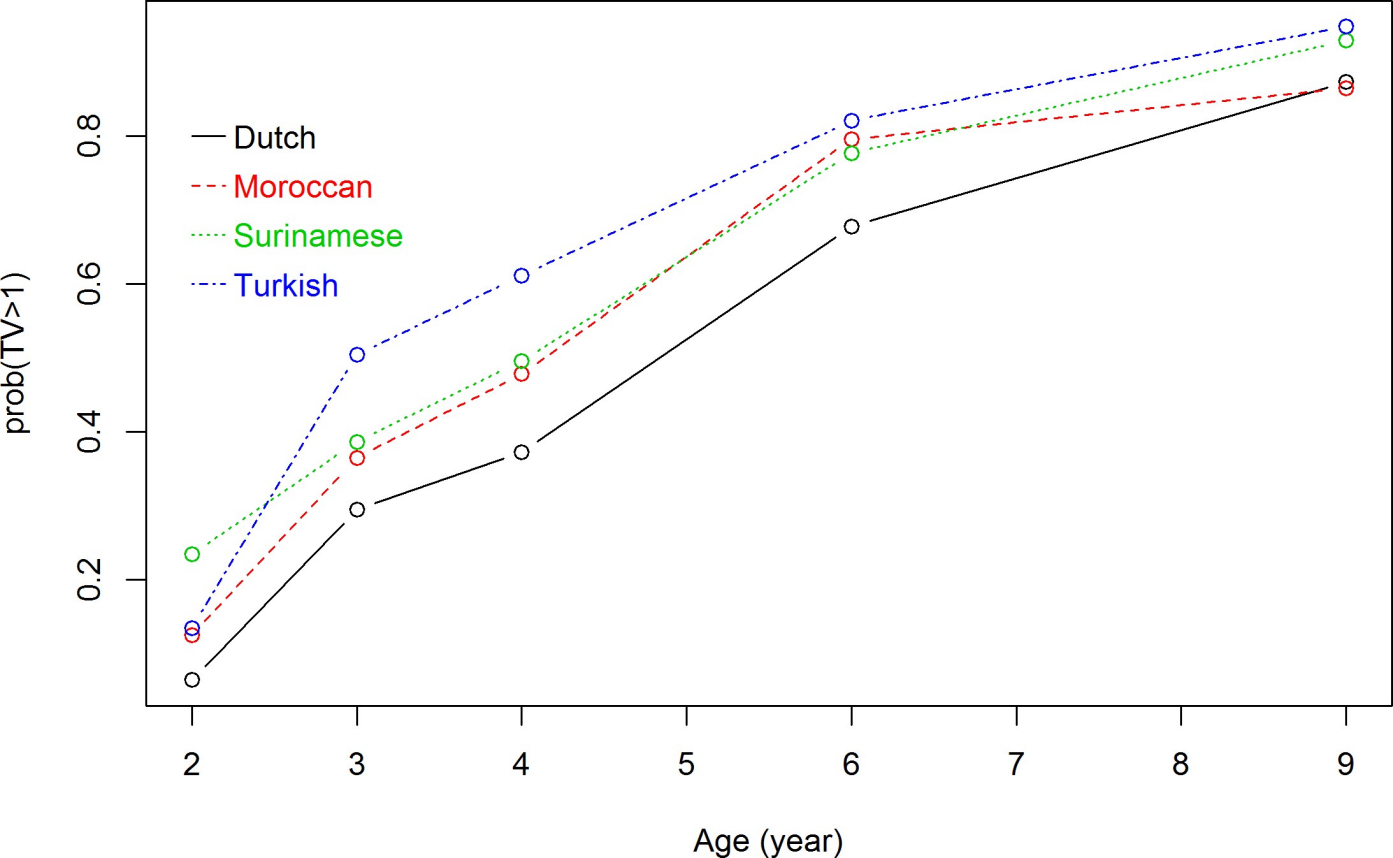
Association between ethnic background and TV viewing time trajectories in children of middle-educated mothers. Results are based on a generalized logistic mixed model and reflect the probability of TV viewing time of >1 hour/day (based on 4,656 measurements) in the first 9 years for children of mothers with middle education level.

**Fig 3:**
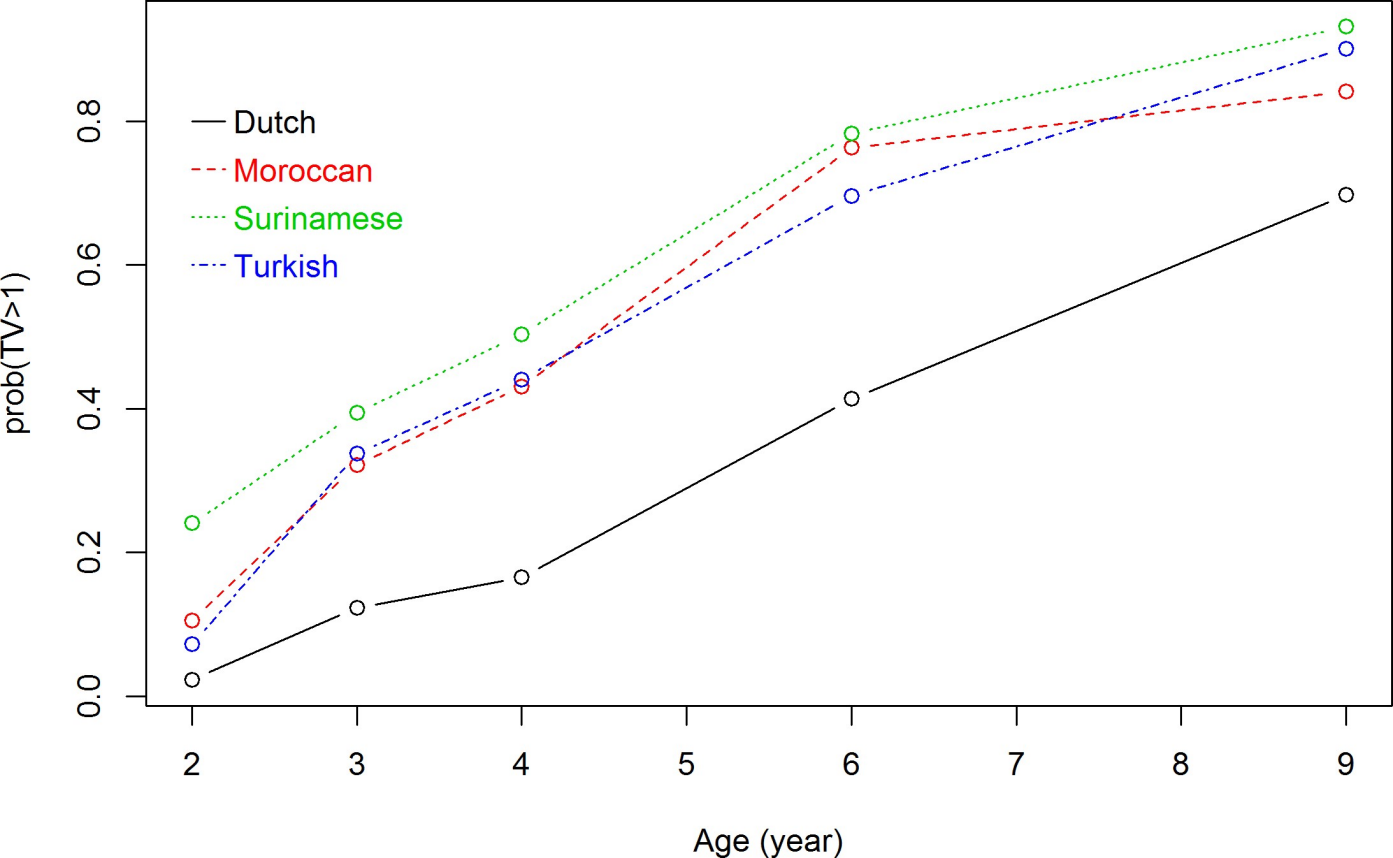
Association between ethnic background and TV viewing time trajectories in children of high-educated mothers. Results are based on a generalized logistic mixed model and reflect the probability of TV viewing time of >1 hour/day (based on 9,276 measurements) in the first 9 years for children of mothers with high education level.

Because at each measuring time point there were missing values for TV viewing time (ranging from 15.1% to 36.4% of the total), for all five time points the total number of measurements of TV viewing time was 16,511. The interaction term between ethnic background and measuring time point, and the interaction between ethnic background and maternal educational level were both significant at p<0.05 and were added to the longitudinal model. The significance of the interaction term between ethnic background and measuring time point indicated that TV viewing trajectories differed significantly between ethnic subgroups. In addition, the significance of the interaction term between ethnic background and maternal educational level indicated that TV viewing trajectories differed significantly at each maternal educational level. Figs 1–3 show the results of the repeated measurement analyses of ethnic background and children’s TV viewing time trajectories in children of respectively high-, middle-, and low-educated mothers. The probability of viewing TV ≥1 hour/day increased over time for all ethnic subgroups. The ethnic differences in probability of watching TV ≥1 hour/day were larger in children of high-educated mothers than in children of low-educated mothers.

## Discussion

This prospective study aimed to assess the trajectories of children’s TV viewing time in an ethnically diverse sample of children from the Netherlands at ages from 2 to 9 years. The study found that in all ethnic subgroups the percentage of children watching TV more than 1 hour/day increased from ages 2 to 9 years. Children with a Turkish, Moroccan, or Surinamese ethnic background had greater odds of exceeding entertainment media guidelines (<1 hour/day) compared to children with a Dutch background, especially at the ages of 4 and 6 years. At these ages, the associations between ethnic background and children’s TV viewing time were moderated by maternal educational level. As we hypothesized, the TV viewing trajectories differed among ethnic subgroups. Apart from that, the disparities in trajectory were different at each maternal educational level.

Cross-sectional analyses revealed that children from other ethnic subgroups had greater odds of watching TV more than 1 hour/ day than children with a Dutch background, especially at school age. This is in line with the findings of previous studies conducted among school-aged children [15, 26]. Haughton et al. reported that among children aged 6–11 years in the United States, the percentage of children engaging in ≤ 2 hours of screen time varied across non-Hispanic whites, Hispanics, non-Hispanic blacks, and Asians [15]. A systematic review reported that 17 out of 21 studies found that ethnic background was associated with TV viewing time among children ≤7 years old. Most of the studies found that ethnic minority group was positively strongly associated with TV viewing time [26]. In our study, association between ethnic background and children’s TV viewing time was not significant at child age 3 years. Before the age of 4 years, only children with a Surinamese background had greater odds of watching TV ≥1 hour/day compared with children with a Dutch background. These findings differ from an earlier report on trajectories of TV viewing time among preschool children from the UK [27]. In that study of children aged 6–36 months, the children of Pakistani mothers spent around 13 minutes more viewing TV a day than the children of white British mothers [27]. However, as the ethnic backgrounds of people in a country reflect that country’s immigration history and culture, the pathways underlying the association between ethnic background and TV viewing time can be expected to differ.

It has been well documented that indicators of family SES (i.e., maternal educational level and net household income) are inversely associated with children’s TV viewing time [21, 26]. Mothers who are not highly educated have been reported to display more positive attitudes about TV viewing, believing that TV programs are instructive and stimulating [28]. Our study too found that maternal educational level had a significant effect modification on ethnic differences in TV viewing time: in the high maternal educational level subgroup, the percentage of children watching TV for more than 1 hour a day was significantly lower in the Dutch children than in the children from the other ethnic backgrounds. Moreover, this ethnic difference was absent among children from low-educated mothers. In addition to cross-sectional analyses, effect modification was observed in longitudinal models evaluating TV viewing time trajectories. In the subgroup with low maternal educational level, children with a Turkish background had lower odds of watching TV more than 1 hour/day compared to children with a Dutch background at all ages we considered. The differences were significant at age 2 years but not thereafter. In the subgroup with a high maternal educational level, children with Moroccan and Turkish backgrounds showed similar TV viewing trajectories. Interestingly, in our study the influence of maternal educational level on ethnic differences is stronger for school-age children than for preschool children. This finding is contrary to our expectation that maternal educational level would have a stronger influence when children are younger and less likely to be influenced by the environment outside the home. Future studies might shed more light on this by studying the interaction effect between maternal educational level and ethnic background in TV viewing time among adolescent children.

It has been consistently reported that parental attitude toward TV viewing and home environment factors are associated with higher children’s TV viewing time [26]. We found that mothers from all ethnic subgroups watched more TV than Dutch mothers at child age 4 years (p<0.017, S4 Table). Apart from parents’ own TV viewing time, parental attitude toward children’s TV viewing (i.e., allowing a TV set in the child’s bedroom) also play an important role in children’s TV viewing behavior. Children from all three non-Dutch ethnic groups more often had a TV set in their bedrooms compared to children with a Dutch background (p<0.001 at all measuring time points, S4 Table). At child age 3 years, only 2.0% of children with a Dutch background had a TV set in their bedroom; the highest percentage, 17.5%, was for children with a Moroccan background. At child age 9 years, 18.6% of children with a Dutch background had a TV set in their bedroom; the highest percentage (34.2%) was found for children with a Surinamese background. The percentage of children exceeding the entertainment media viewing guidelines (<1 hour/day) was higher among children with a TV set in their bedroom than among children without a TV set in their bedroom (p<0.001 at all measuring time points, S5 Table). These results suggest that in addition to parental education and income, other culturally influenced environmental factors contribute to ethnic differences in children’s TV viewing time.

Some longitudinal studies have reported an association between higher TV viewing time and child obesity [29, 30]. It is therefore important to provide policy makers and researchers with information regarding the TV viewing time of population groups. Our findings from trajectory analyses highlight the time points that may be suitable for targeted intervention programs. Interventions should also take into account parents’ cultural beliefs and values, as these may impact children’s health-related behavior.

## Methodological considerations

The strengths of this study include the large sample of children from different ethnic backgrounds and the availability of data on repeatedly measured TV viewing time at five time points over the first nine years of life. The longitudinal design enabled trajectories to be plotted and the identification of a key time point and population groups that may be suitable for targeted intervention programs. Several limitations of this study should be considered when interpreting the results. First, about 19% of the participants who were eligible for inclusion in this study based on their ethnic background were excluded from the analyses because data were not available on TV viewing time for all five time points. Nonresponse analyses showed that data for all five time points were more often missing for children from ethnic minority groups and low SES groups. Selection bias may have occurred if the association between ethnic background and TV viewing time differed between participants and nonparticipants. Second, in this study the parental country of birth plays a central role in the definition of child/maternal ethnic background. The definition we use implies ethnic background based on the ethnic origin of the study population, not on their nationality and/or ethnic identity. Future studies are recommended to take more components of ethnic background into account. Third, as information on children’s TV viewing time was derived from parent-reported questionnaires, socially desirable answers (i.e., the over-reporting of favorable behaviors) cannot be excluded. Furthermore, information bias in the TV viewing time may have occurred due to the use of different items in questionnaires at each age (see S1 Table). We used the number of days of TV viewing at age 4 years to estimate daily TV viewing time at ages 2 and 3 years. This may have introduced information bias. Children at ages 2 and 3 may have watched TV on more or fewer days per week than at age 4 years. Furthermore, sensitivity analyses using TV viewing time dichotomized at 2 hours per day were performed on data at ages 6 and 9 years. The results of the cross-sectional analyses were comparable to the previous analyses, although the effect estimates (ORs) were larger (see S2 Table). Sensitivity analyses were not performed on data at ages 2 to 4 years because it is always recommended that this age group watches TV for no more than 1 h/day. Also, in our study population the sample of children watching TV for more than 2 hours per day is relatively small. None of the children watched TV ≥2 hours/day at age 2 years (Table 1).

## Conclusions

To sum up: the percentage of children watching TV more than 1 hour/day increased from age 2 to 9 years for these children with Dutch, Turkish, Moroccan, and Surinamese backgrounds. The ethnic differences in TV viewing time were present at all measuring time points. The children’s TV viewing trajectories differed among ethnic subgroups at each maternal educational level. The gap between children with a Dutch background and children with a different ethnic background was larger in high maternal educational subgroups. Our results suggest that interventions intended to reduce TV viewing time should target children of non-Dutch ethnic groups and their parents when the children are at an early age. Future studies are recommended to follow adolescents’ TV viewing trajectories and the association with ethnic background.

## Supporting information

S1 Appendix Flowchart of participants included for analysis. (DOCX)

S1 Table Questionnaire items and calculation of the TV viewing time. (DOCX)

S2 Table Associations of ethnic background with TV viewing time (≥ 2 hours/day) according to maternal educational level, at child ages 6 and 9 years (n = 4,833). (DOCX)

S3 Table Associations of ethnic background with TV viewing time according to paternal educational level at each age (n = 4,833). (DOCX)

S4 Table Parental attitude towards children’s TV viewing time according to ethnic background (N = 4,833). (DOCX)

S5 Table Children’s TV viewing time according to TV set in child’s bedroom. (DOCX)

S1 Fig Association between ethnic background and TV viewing time trajectories in children of low-educated mothers (confidence interval bars included). Results are based on a generalized logistic mixed model and reflect the probability of TV viewing time of >1 hour/day (based on 2,579 measurements) in the first 9 years for children of mothers with low education level.
(TIFF)

S2 Fig Association between ethnic background and TV viewing time trajectories in children of middle-educated mothers (confidence interval bars included). Results are based on a generalized logistic mixed model and reflect the probability of TV viewing time of >1 hour/day (based on 4,656 measurements) in the first 9 years for children of mothers with middle education level.
(TIFF)

S3 Fig Association between ethnic background and TV viewing time trajectories in children of high-educated mothers (confidence interval bars included). Results are based on a generalized logistic mixed model and reflect the probability of TV viewing time of >1 hour/day (based on 9,276 measurements) in the first 9 years for children of mothers with high education level.
(TIFF)

## Abbreviations

SES: socioeconomic status
TV: television
ORs: Odds ratios
CI: confidence intervals

## References

[1] Carson V, Hunter S, Kuzik N, Gray CE, Poitras VJ, Chaput JP, et al Systematic review of sedentary behaviour and health indicators in school-aged children and youth: an update. Appl Physiol Nutr Metab. 2016;41(6 Suppl 3):S240–65. 10.1139/apnm-2015-0630 27306432 10.1139/apnm-2015-0630

[2] Department of Health. Australia’s Physical Activity and Sedentary Behaviour Guidelines. 2014.

[3] Tremblay MS, Leblanc AG, Janssen I, Kho ME, Hicks A, Murumets K, et al Canadian sedentary behaviour guidelines for children and youth. Appl Physiol Nutr Metab. 2011;36(1):59–64; 5–71. 10.1139/H11-012 21326378 10.1139/H11-012

[4] Council On Communications Media. Media and Young Minds. Pediatrics. 2016;138(5). 10.1542/peds.2016-2591 27940793

[5] Colley RC, Garriguet D, Adamo KB, Carson V, Janssen I, Timmons BW, et al Physical activity and sedentary behavior during the early years in Canada: a cross-sectional study. Int J Behav Nutr Phys Act. 2013;10:54 10.1186/1479-5868-10-54 23642258 10.1186/1479-5868-10-54 PMC3655822

[6] Verloigne M, Loyen A, Van Hecke L, Lakerveld J, Hendriksen I, De Bourdheaudhuij I, et al Variation in population levels of sedentary time in European children and adolescents according to cross-European studies: a systematic literature review within DEDIPAC. Int J Behav Nutr Phys Act. 2016;13:69 10.1186/s12966-016-0395-5 27350043 10.1186/s12966-016-0395-5 PMC4924322

[7] Arundell L, Fletcher E, Salmon J, Veitch J, Hinkley T. A systematic review of the prevalence of sedentary behavior during the after-school period among children aged 5–18 years. Int J Behav Nutr Phys Act. 2016;13:93 10.1186/s12966-016-0419-1 27549588 10.1186/s12966-016-0419-1 PMC4994288

[8] Pearson N, Salmon J, Campbell K, Crawford D, Timperio A. Tracking of children's body-mass index, television viewing and dietary intake over five-years. Prev Med. 2011;53(4–5):268–70. 10.1016/j.ypmed.2011.07.014 21820008 10.1016/j.ypmed.2011.07.014

[9] Janssen X, Mann KD, Basterfield L, Parkinson KN, Pearce MS, Reilly JK, et al Development of sedentary behavior across childhood and adolescence: longitudinal analysis of the Gateshead Millennium Study. Int J Behav Nutr Phys Act. 2016;13(1):88. 27484336 10.1186/s12966-016-0413-7 PMC4971697

[10] Rideout VJ, Foehr UG, Roberts DF. Generation M2: Media in the Lives of 8- to 18-Year-Olds. 2010.

[11] Sisson SB, Broyles ST, Baker BL, Katzmarzyk PT. Television, reading, and computer time: correlates of school-day leisure-time sedentary behavior and relationship with overweight in children in the U.S. J Phys Act Health. 2011;8 Suppl 2:S188–97. 21918232

[12] Karlsen S, Morris S, Kinra S, Vallejo-Torres L, Viner RM. Ethnic variations in overweight and obesity among children over time: findings from analyses of the Health Surveys for England 1998–2009. Pediatr Obes. 2014;9(3):186–96. 10.1111/j.2047-6310.2013.00159.x 23554401 10.1111/j.2047-6310.2013.00159.x PMC4171811

[13] Kimbro RT, Brooks-Gunn J, McLanahan S. Racial and ethnic differentials in overweight and obesity among 3-year-old children. Am J Public Health. 2007;97(2):298–305. 10.2105/AJPH.2005.080812 17194857 10.2105/AJPH.2005.080812 PMC1781385

[14] Dijkshoorn H, Nicolaou M, Ujcic-Voortman JK, Schouten GM, Bouwman-Notenboom AJ, Berns MP, et al Overweight and obesity in young Turkish, Moroccan and Surinamese migrants of the second generation in the Netherlands. Public Health Nutr. 2014;17(9):2037–44. 10.1017/S1368980013002322 24053886 10.1017/S1368980013002322 PMC11108712

[15] Haughton CF, Wang ML, Lemon SC. Racial/Ethnic Disparities in Meeting 5-2-1-0 Recommendations among Children and Adolescents in the United States. J Pediatr. 2016;175:188–94 e1. 10.1016/j.jpeds.2016.03.055 27112040 10.1016/j.jpeds.2016.03.055 PMC5988361

[16] Goh SN, Teh LH, Tay WR, Anantharaman S, van Dam RM, Tan CS, et al Sociodemographic, home environment and parental influences on total and device-specific screen viewing in children aged 2 years and below: an observational study. BMJ Open. 2016;6(1):e009113 10.1136/bmjopen-2015-009113 26810995 10.1136/bmjopen-2015-009113 PMC4735142

[17] Wijtzes AI, Jansen W, Jaddoe VW, Moll HA, Tiemeier H, Verhulst FC, et al Ethnic background and television viewing time among 4-year-old preschool children: the generation R study. J Dev Behav Pediatr. 2013;34(2):63–71. 10.1097/DBP.0b013e31827b163a 23369955 10.1097/DBP.0b013e31827b163a

[18] Kooijman MN, Kruithof CJ, van Duijn CM, Duijts L, Franco OH, van IMH, et al The Generation R Study: design and cohort update 2017. Eur J Epidemiol. 2016;31(12):1243–64. 10.1007/s10654-016-0224-9 28070760 10.1007/s10654-016-0224-9 PMC5233749

[19] Jaddoe VW, van Duijn CM, van der Heijden AJ, Mackenbach JP, Moll HA, Steegers EA, et al The Generation R Study: design and cohort update 2010. Eur J Epidemiol. 2010;25(11):823–41. 10.1007/s10654-010-9516-7 20967563 10.1007/s10654-010-9516-7 PMC2991548

[20] Statistics Netherlands. Annual report on integration 2012 Summary2012. Available from: https://www.cbs.nl/en-gb/publication/2012/51/annual-report-on-integration-2012-summary.

[21] Yang-Huang J, van Grieken A, Moll HA, Jaddoe VWV, Wijtzes AI, Raat H. Socioeconomic differences in children's television viewing trajectory: A population-based prospective cohort study. PLoS One. 2017;12(12):e0188363 10.1371/journal.pone.0188363 29211770 10.1371/journal.pone.0188363 PMC5718560

[22] Statistics Netherlands. Standaard Onderwijsindeling 2003: Voorburg/Heerlen 2004.

[23] Netherlands Bureau for Economic Policy Analysis. updated May 9, 2017. Available from: http://www.cpb.nl.

[24] Mickey RM, Greenland S. The impact of confounder selection criteria on effect estimation. Am J Epidemiol. 1989;129(1):125–37. 2910056 10.1093/oxfordjournals.aje.a115101

[25] Greenland S, Finkle WD. A critical look at methods for handling missing covariates in epidemiologic regression analyses. Am J Epidemiol. 1995;142(12):1255–64. 7503045 10.1093/oxfordjournals.aje.a117592

[26] Hoyos Cillero I, Jago R. Systematic review of correlates of screen-viewing among young children. Prev Med. 2010;51(1):3–10. 10.1016/j.ypmed.2010.04.012 20417227 10.1016/j.ypmed.2010.04.012

[27] Barber SE, Kelly B, Collings PJ, Nagy L, Bywater T, Wright J. Prevalence, trajectories, and determinants of television viewing time in an ethnically diverse sample of young children from the UK. Int J Behav Nutr Phys Act. 2017;14(1):88 10.1186/s12966-017-0541-8 28683801 10.1186/s12966-017-0541-8 PMC5501260

[28] Valerio M, Amodio P, Dal Zio M, Vianello A, Zacchello GP. The use of television in 2- to 8-year-old children and the attitude of parents about such use. Arch Pediatr Adolesc Med. 1997;151(1):22–6. 9006524 10.1001/archpedi.1997.02170380026004

[29] Rey-Lopez JP, Vicente-Rodriguez G, Biosca M, Moreno LA. Sedentary behaviour and obesity development in children and adolescents. Nutr Metab Cardiovasc Dis. 2008;18(3):242–51. 10.1016/j.numecd.2007.07.008 18083016 10.1016/j.numecd.2007.07.008

[30] Hancox RJ, Poulton R. Watching television is associated with childhood obesity: but is it clinically important? Int J Obes (Lond). 2006;30(1):171–5. 10.1038/sj.ijo.0803071 16158085

