# Supplementary material for: Ethnic background and children’s television viewing trajectories: The Generation R Study
Source: PLoS One. 2018 Dec 14;13(12):e0209375. doi: 10.1371/journal.pone.0209375 (PMC6294372; doi:10.1371/journal.pone.0209375)
Supplement: S1 Appendix — (DOCX) [file pone.0209375.s001.docx]

Children for follow up studies 0-4 years or 6, 9 years onwards of The Generation R Study

**N=9162**

Children participate in 0-4 years or 6, 9 years onwards of The Generation R Study

**N=6497**

N=2665

Excluded: small numbers of ethnicities

Children with at least one TV viewing time data at age 24 months, 36 months, 48 months, 6 years and 9 years

**N=5288**

Population for analysis

**N=4833**

Dutch: N=3561

Turkish: N=498

Moroccan: N=317

Surinamese: N=457

N=1209

Excluded: missing data on all 5 time points of TV viewing time

N=455

Excluded:

Second child of the same mother: N=446

Third child of the same mother: N=9

**S1 Appendix. Flowchart of participants included for analysis**
