# Supplementary material for: Ethnic background and children’s television viewing trajectories: The Generation R Study
Source: PLoS One. 2018 Dec 14;13(12):e0209375. doi: 10.1371/journal.pone.0209375 (PMC6294372; doi:10.1371/journal.pone.0209375)
Supplement: S1 Table — (DOCX) [file pone.0209375.s002.docx]

**S2 Table. Questionnaire items and calculation of the TV viewing time**

| **Age (measuring time point)** | **Question** | **Response Scale**  **(assigned value)** | **Label** | **TV viewing time** |
| --- | --- | --- | --- | --- |
| 2 (1) | How long on average, does your child watch TV per day during the week? | Never (0), less than 0.5 hours (0.25), 0.5 hours to 1 hour (0.75), more than 1 hour (1.5) | A_2_ | TVtime@2 = (A_2_ * 5 + B_2_ * 2) / 7 * D_4_/ 7 ➀ |
|  | How long on average, does your child watch TV per day during the weekend? | Less than 1 hour (0.5), 1 hour to 2 hours (1.5), more than 2 hours (2.5) | B_2_ |  |
| 3 (2) | How much time has your child been occupied with watching television in the last month (On most weekdays)? | None or less than 30 minutes per day (0.25), 30 minutes to 1 hour per day (0.75), 1 to 2 hours per day (1.5), 2 to 3 hours per day (2.5), more than 3 hours per day (3.5) | A_3_ | TVtime@3 = (A_3_*5 + B_3_*2)/7* D_4_/7 ➀ |
|  | How much time has your child been occupied with watching television in the last month (During the weekend)? | None or less than 30 minutes per day (0.25), 30 minutes to 1 hour per day (0.75), 1 to 2 hours per day (1.5), 2 to 3 hours per day (2.5), more than 3 hours per day (3.5) | B_3_ |  |
| 4 (3) | How many days per week does your child watch TV/Video/DVD?➁ | Never, less than 1 day per week (0), 1 day per week (1), 2 days per week (2), 3 days per week (3), 4 days per week (4), 5 days per week (5), 6 days per week (6) | D_4_ | TVtime@4 = D_4_ * T_4_/ 7 |
|  | How long does your child generally watch TV/Video/DVD per day for? | Less than half an hour per day (0.25), between half an hour and 1 hour per day (0.75), 1 to 2 hours per day (1.5), 2 to 3 hours per day (2.5), more than 3 hours per day (3.5) | T_4_ |  |
| 6 (4) and 9 (5) | On average, how many weekdays per week does your child watch television/video/DVD? | Never on weekdays (0), 1 day (1), 2 days (2), 3 days (3), 4 days (4), every weekday (5) | D_6i_, D_9i_ | TVtime@6 = [(A_6i_ + A_6j_ + A_6m_) * D_6i_ + (B_6i_ + B_6j_ + B_6m_) * D_6j_] / 7  TVtime@9 = [(A_9i_ + A_9j_ + A_9m_) * D_9i_ + (B_9i_ + B_9j_ + B_9m_) * D_9j_] / 7 |
|  | On average, how many weekend days per week does your child watch television/video/DVD? | Never in the weekend (0), 1 day in the weekend (1),2 days in the weekend (2) | D_6j_, D_9j_ |  |
|  | On the days that your child watches television/video/DVD, how long does he watch, on average?  Differentiate here between weekdays and week-ends.  - Weekday mornings  - Weekday afternoons  - Weekday evenings after dinner  - weekend mornings  - Weekend afternoons  - Weekend evenings after dinner | Never (0), less than 30 minutes (0.25), 30-60 minutes (0.75), 1-2 hours (1.5), 2-3 hours (2.5), 3-4 hours (3.5) | A_6i_, A_6j_, A_6m_,  B_6i_, B_6j_, B_6m_  A_9i_, A_9j_, A_9m_,  B_9i_, B_9j_, B_9m_ |  |

➀ At age 2 and 3 years we used the number of days of TV viewing at age 4 years when calculating the average TV time per day.

➁At age 4, 51% of parents indicated that their children watched TV seven days per week.
