# Supplementary material for: Ethnic background and children’s television viewing trajectories: The Generation R Study
Source: PLoS One. 2018 Dec 14;13(12):e0209375. doi: 10.1371/journal.pone.0209375 (PMC6294372; doi:10.1371/journal.pone.0209375)
Supplement: S2 Table — (DOCX) [file pone.0209375.s003.docx]

**S2 Table. Associations of ethnic background with TV viewing time (≥ 2 hours/day) according to maternal educational level at age 6 and 9 years (n=4,833)**

|  | **Ethnic background** | **Measuring time point** | |
| --- | --- | --- | --- |
|  |  | **Age 6 years** | **Age 9 years** |
|  |  | **OR (95%CI)** | **OR (95%CI)** |
| Total study population^a^ | Dutch | 1.00 | 1.00 |
|  | Turkish | **2.49 (1.90, 3.26)** | **1.84 (1.35, 2.52)** |
|  | Moroccan | **2.67 (1.93, 3.71)** | **1.60 (1.12, 2.29)** |
|  | Surinamese | **3.27 (2.53, 4.23)** | **2.76 (2.09, 3.65)** |
| **Maternal educational level^b^** | **Ethnic background** | **Measuring time point** | |
|  |  | **Age 6 years** | **Age 9 years** |
|  |  | **OR (95%CI)** | **OR (95%CI)** |
| High | Dutch | 1.00 | 1.00 |
|  | Turkish | **5.28 (2.47, 11.29)** | **5.17 (2.49, 10.74)** |
|  | Moroccan | 2.42 (0.97, 6.00) | **4.68 (2.20, 9.98)** |
|  | Surinamese | **6.34 (3.71, 10.82)** | **5.83 (3.45, 9.83)** |
| Middle | Dutch | 1.00 | 1.00 |
|  | Turkish | **2.49 (1.62, 3.81)** | 1.61 (0.99, 2.62) |
|  | Moroccan | **2.67 (1.55, 4.60)** | 1.38 (0.77, 2.48) |
|  | Surinamese | **2.78 (1.92, 4.03)** | **2.26 (1.54, 3.32)** |
| Low | Dutch | 1.00 | 1.00 |
|  | Turkish | **1.89 (1.30, 2.75)** | 1.09 (0.67, 1.77) |
|  | Moroccan | **2.39 (1.50, 3.82)** | 0.84 (0.48, 1.49) |
|  | Surinamese | **2.47 (1.55, 3.94)** | 1.58 (0.90, 2.78) |

Table is based on imputed dataset. Bold print indicates statistical significance. Values represent odds ratios and 95% confidence intervals derived from multiple logistic regression analyses.

^a^ Models were adjusted for child’s exact age, maternal educational level and net household income

^b^ Models were adjusted for child’s exact age and net household income.
