# Supplementary material for: Ethnic background and children’s television viewing trajectories: The Generation R Study
Source: PLoS One. 2018 Dec 14;13(12):e0209375. doi: 10.1371/journal.pone.0209375 (PMC6294372; doi:10.1371/journal.pone.0209375)
Supplement: S3 Table — (DOCX) [file pone.0209375.s004.docx]

**S3 Table. Associations of ethnic background with TV viewing time according to paternal educational level at each age (n=4,833)**

| **Paternal educational level** | **Ethnic background** | **Measuring time point** | | | | |
| --- | --- | --- | --- | --- | --- | --- |
|  |  | **Age 2 years** | **Age 3 years** | **Age 4 years** | **Age 6 years** | **Age 9 years** |
|  |  | **OR (95%CI)** | **OR (95%CI)** | **OR (95%CI)** | **OR (95%CI)** | **OR (95%CI)** |
| High | Dutch | 1.00 | 1.00 | 1.00 | 1.00 | 1.00 |
|  | Turkish | **3.07 (1.35, 7.00)** | **2.43 (1.23, 4.82)** | **2.23 (1.21, 4.11)** | **2.68 (1.49, 4.82)** | **2.53 (1.03, 6.25)** |
|  | Moroccan | 3.19 (0.65, 15.60) | 2.07 (0.49, 8.83) | 2.99 (0.98, 9.12) | **2.91 (1.12, 7.55)** | 1.22 (0.45, 3.29) |
|  | Surinamese | **6.61 (3.20, 13.66)** | **4.02 (2.11, 7.68)** | **2.21 (1.22, 3.99)** | 1.62 (0.90, 2.93) | **4.53 (1.59, 12.92)** |
| Middle | Dutch | 1.00 | 1.00 | 1.00 | 1.00 | 1.00 |
|  | Turkish | 1.26 (0.51, 3.09) | 1.00 (0.43, 2.32) | 1.38 (0.69, 2.74) | 1.96 (1.00, 3.84) | 2.21 (0.74, 6.60) |
|  | Moroccan | 1.20 (0.32, 4.41) | **5.18 (1.39, 19.28)** | 1.72 (0.63, 4.67) | 3.18 (0.89, 11.37) | 2.31 (0.51, 10.56) |
|  | Surinamese | 2.05 (0.97, 4.36) | **4.02 (2.11, 7.68)** | 1.67 (0.89, 3.14) | **2.20 (1.21, 4.01)** | **2.51 (1.03, 6.10)** |
| Low | Dutch | 1.00 | 1.00 | 1.00 | 1.00 | 1.00 |
|  | Turkish | 0.40 (0.15, 1.08) | 0.88 (0.45, 1.72) | 1.06 (0.59, 1.92) | 0.70 (0.40, 1.21) | 0.97 (0.38, 2.48) |
|  | Moroccan | 0.54 (0.15, 1.91) | 1.09 (0.44, 2.74) | 1.62 (0.71, 3.68) | 1.45 (0.57, 3.69) | 1.28 (0.36, 4.49) |
|  | Surinamese | 1.71 (0.74, 3.97) | 0.59 (0.25, 1.39) | 1.12 (0.56, 2.20) | 1.42 (0.73, 2.75) | 1.30 (0.52, 3.23) |

Table is based on imputed dataset. Bold print indicates statistical significance. Values represent odds ratios and 95% confidence intervals derived from multiple logistic regression analyses.

Models were adjusted for child’s exact age and net household income.
