# Supplementary material for: Ethnic background and children’s television viewing trajectories: The Generation R Study
Source: PLoS One. 2018 Dec 14;13(12):e0209375. doi: 10.1371/journal.pone.0209375 (PMC6294372; doi:10.1371/journal.pone.0209375)
Supplement: S4 Table — (DOCX) [file pone.0209375.s005.docx]

**S4 Table. Parental attitude towards children’s TV viewing time according to ethnic background (N=4,833)**

|  |  | Ethnic background | | | | P-value^b^ |
| --- | --- | --- | --- | --- | --- | --- |
|  |  | Dutch | Turkish | Moroccan | Surinamese |  |
| TV set in child’s bedroom^a^ | Age 3 years | 42 (1.8) | 28 (11.2) | 24 (16.6) | 17 (9.1) | <0.001 |
|  | Age 6 years | 330 (12.0) | 122 (31.4) | 77 (31.2) | 88 (28.9) | <0.001 |
|  | Age 9 years | 407 (17.6) | 72 (33.0) | 43 (26.2) | 82 (36.0) | <0.001 |
| Parents’ TV viewing time (age 4 years) | A lot | 248 (10.4) | 37 (14.3) | 27 (16.7) | 34 (16.8) | 0.003 |
|  | Neither a lot nor a little | 1180 (49.7) | 139 (53.7) | 81 (50.0) | 101 (50.0) |  |
|  | A little | 947 (39.9) | 83 (32.0) | 54 (33.3) | 67 (33.2) |  |

Table is based on non-imputed dataset.

^a^ Values are percentages by different ethnic background.

^b^ P-values are calculated by Chi-square test for categorical variables
