# Supplementary material for: Ethnic background and children’s television viewing trajectories: The Generation R Study
Source: PLoS One. 2018 Dec 14;13(12):e0209375. doi: 10.1371/journal.pone.0209375 (PMC6294372; doi:10.1371/journal.pone.0209375)
Supplement: S5 Table — (DOCX) [file pone.0209375.s006.docx]

**S5 Table. Children’s TV viewing time according to TV set in child’s bedroom**

|  |  | TV viewing time | | P-value^a^ |
| --- | --- | --- | --- | --- |
|  |  | ≥ 1 hour/day | < 1 hour/day |  |
| TV set in child’s bedroom | Age 3 years | 70 (7.4) | 38 (1.8) | <0.001 |
|  | Age 6 years | 505 (21.4) | 191 (11.0) | <0.001 |
|  | Age 9 years | 598 (25.1) | 99 (11.1) | <0.001 |

Table is based on non-imputed dataset.

^a^ P-values are calculated by Chi-square test for categorical variables
