# Supplementary figures and images for: Ethnic background and children’s television viewing trajectories: The Generation R Study
Source: PLoS One. 2018 Dec 14;13(12):e0209375. doi: 10.1371/journal.pone.0209375 (PMC6294372; doi:10.1371/journal.pone.0209375)

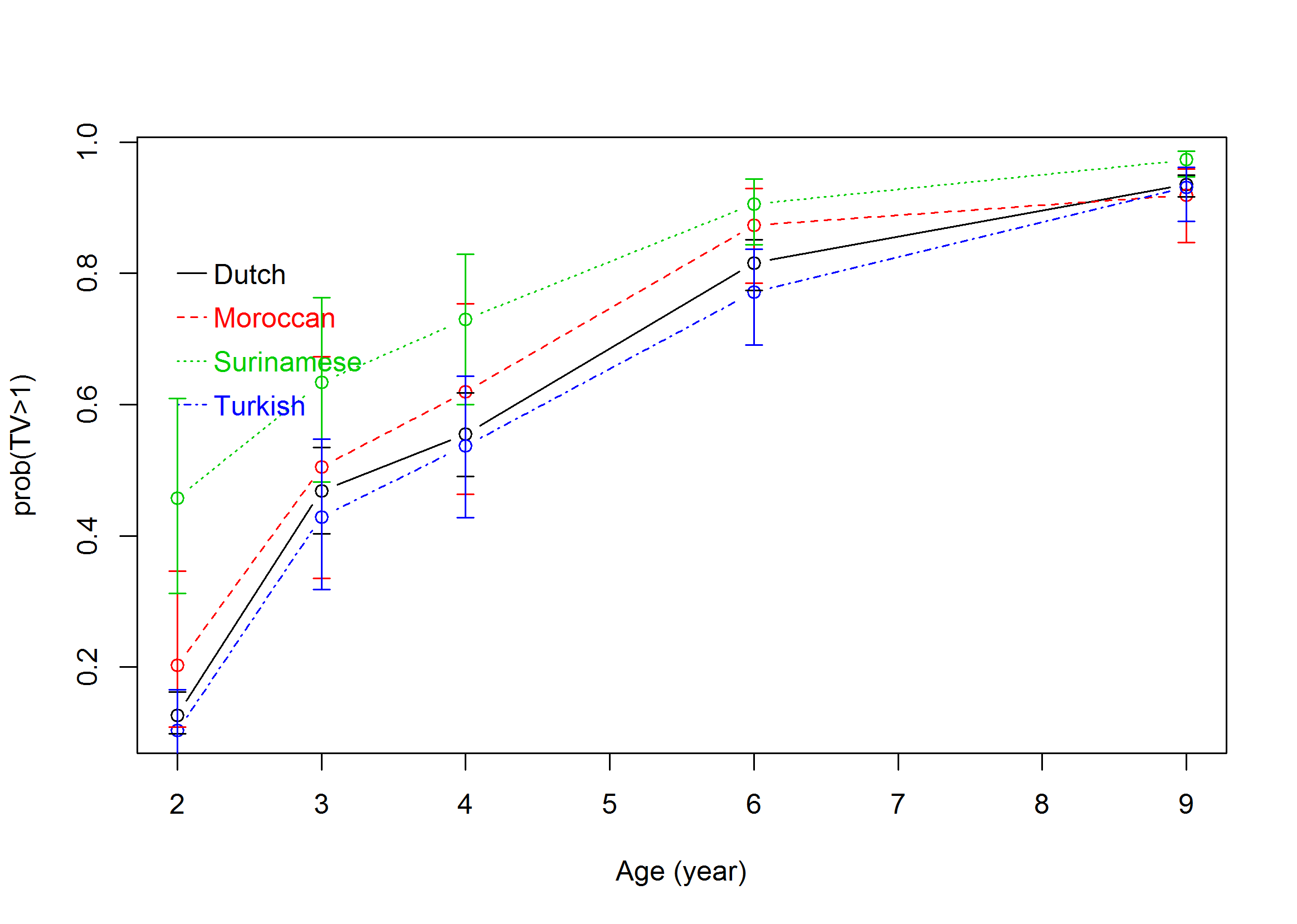

Supplement: S1 Fig — Results are based on a generalized logistic mixed model and reflect the probability of TV viewing time of >1 hour/day (based on 2,579 measurements) in the first 9 years for children of mothers with low education level. (TIFF) [file pone.0209375.s007.tiff]

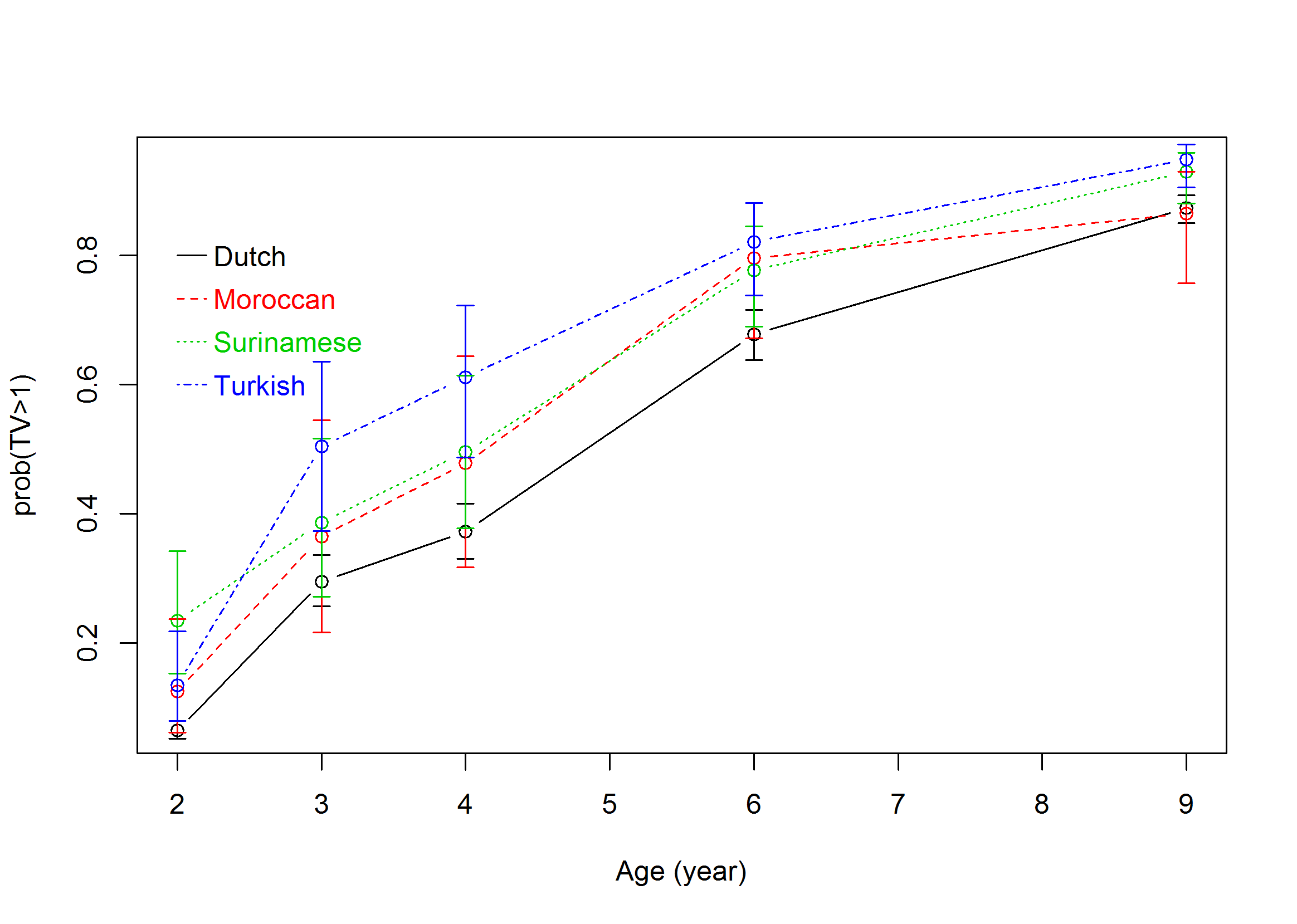

Supplement: S2 Fig — Results are based on a generalized logistic mixed model and reflect the probability of TV viewing time of >1 hour/day (based on 4,656 measurements) in the first 9 years for children of mothers with middle education level. (TIFF) [file pone.0209375.s008.tiff]

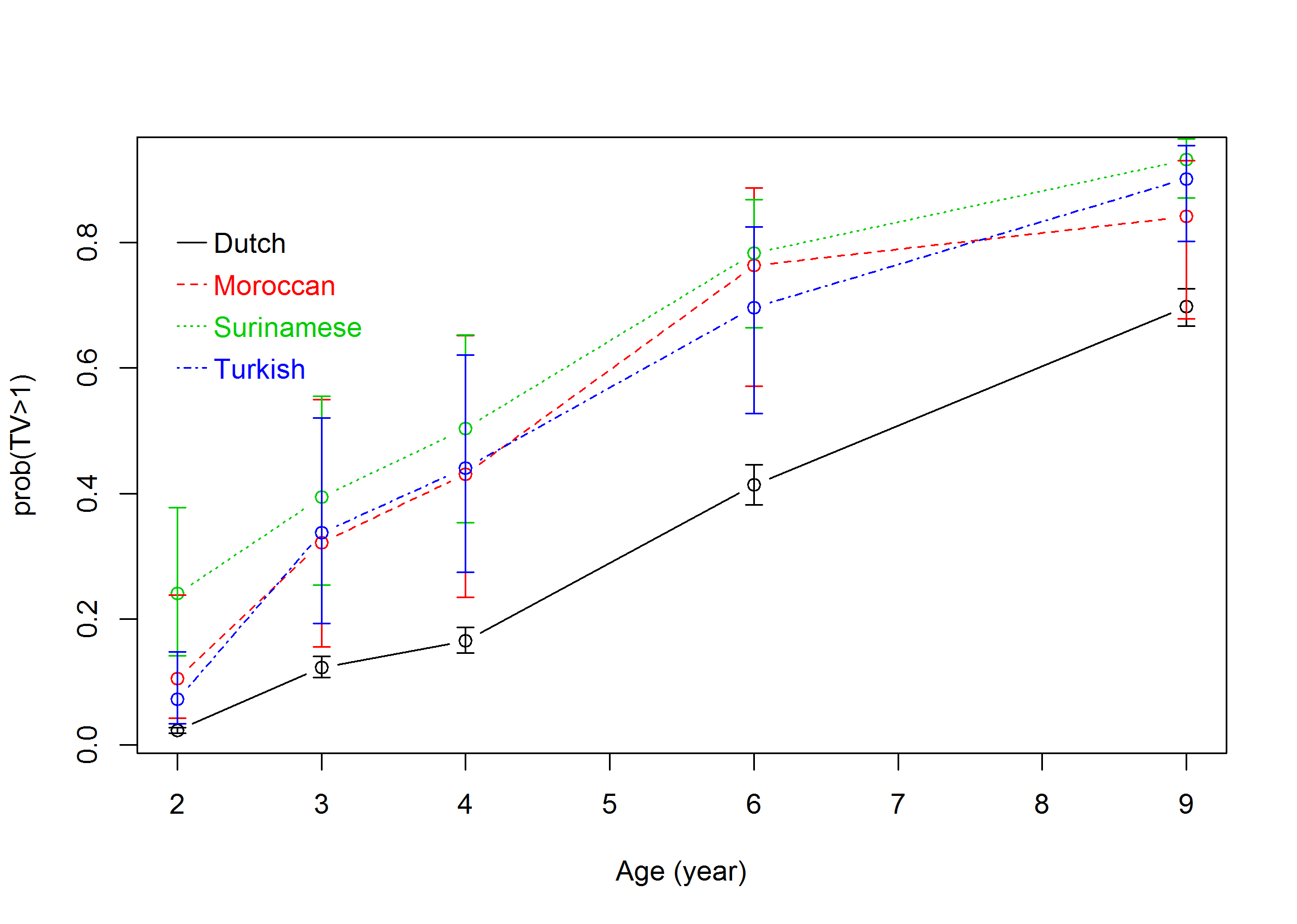

Supplement: S3 Fig — Results are based on a generalized logistic mixed model and reflect the probability of TV viewing time of >1 hour/day (based on 9,276 measurements) in the first 9 years for children of mothers with high education level. (TIFF) [file pone.0209375.s009.tiff]
